# Intradiscal injection of simvastatin retards progression of intervertebral disc degeneration induced by stab injury

**DOI:** 10.1186/ar2861

**Published:** 2009-11-13

**Authors:** Huina Zhang, Lin Wang, Jun Beom Park, Paul Park, Victor C Yang, Scott J Hollister, Frank La Marca, Chia-Ying Lin

**Affiliations:** 1 Spine Research Laboratory, Department of Neurosurgery, University of Michigan Medical School, 1500 E. Medical Center Drive, Ann Arbor, Michigan 48109, USA; 2 Department of Biomedical Engineering, University of Michigan, 2200 Bonisteel Blvd., Ann Arbor, Michigan 48109-2099, USA; 3 Department of Pharmaceutical Sciences, College of Pharmacy, University of Michigan, 428 Church Street, Ann Arbor, MI 48109-1065, USA

## Abstract

**Introduction:**

Earlier work indicates that the cholesterol-lowering drug, simvastatin, is anabolic to chondrogenic expression of rat intervertebral disc (IVD) cells, which suggests a potential role for simvastatin in IVD regeneration. In this study, we expand on our earlier work to test the effectiveness of simvastatin on disc degeneration utilizing a rat tail disc degeneration model.

**Methods:**

30 rats that underwent 21 G needle-puncture at rat tail discs were injected with simvastatin-loaded poly(ethylene glycol)-poly(lactic acid-co-glycolic acid)-poly(ethylene glycol) (PEG-PLGA-PEG) gel (5 mg/ml) or vehicle control at 4 weeks after needle injury. All animals were sacrificed 2 weeks after simvastatin injection. Bone morphogenetic protein-2 (BMP-2), aggrecan, collagen type II, and collagen type I messenger ribonucleic acid (mRNA) expression in the rat nucleus pulposus (NP) were measured by real-time polymerase chain reaction (PCR). *In vivo *magnetic resonance imaging (MRI) was performed to monitor changes in disc degeneration. Rat discs were also assessed by histology using hematoxylin and eosin (H&E) and safranin O staining. In addition, the NP weight, glycosaminoglycan (sGAG) and DNA content were also measured.

**Results:**

A single dose of simvastatin loaded in thermo-sensitive PEG-PLGA-PEG gel injected into the NP had the trend to increase aggrecan expression and sGAG content, and significantly increased mRNA levels of BMP-2, collagen type II, and the differentiation index (the ratio of collagen type II to collagen type I). The decreased NP weight, T2 intensity, as well as MRI index in the rat tail discs induced by needle puncture were significantly reversed after 2 weeks of simvastatin treatment. In addition, simvastatin treatment also improved histological changes induced by needle puncture.

**Conclusions:**

A single injection of simvastatin loaded in PEG-PLGA-PEG gel into rat tail discs had the potential to retard or regenerate the degenerative disc.

## Introduction

Intervertebral disc (IVD) degeneration is the leading etiological contributor to low back pain and other disc disorders, which can cause significant socioeconomic impact. Disc degeneration often starts with cellular and biochemical changes in the nucleus pulposus (NP) and annulus fibrosus (AF), resulting in an imbalance between anabolism and catabolism of disc tissues. NP, the main constituent of the IVD, plays a major role in maintaining normal function of the IVD. As a consequence, any cellular, biological, or biochemical changes in the NP ultimately deteriorate disc function, leading to disc degeneration.

Despite its high prevalence, the current treatments for degenerative disc disease, including steroid injection [1,2], physical therapy [3,4], intradiscal electrothermal therapy [5,6] and invasive-surgical intervention, are limited to ameliorating symptoms and do not address the etiological problem [7]. With the recent advances in recombinant therapeutic proteins [8,9] biological repair or regeneration of the degenerative IVD has been advocated. This novel treatment option appears promising, because it facilitates synthesis of matrix molecules that compose the IVD structure, and may also help prevent matrix degradation and/or cell death to detain the progression of disc degeneration.

Many growth factors, such as the bone morphogenetic protein (BMP) family (BMP-2, Osteogenic protein-1 (OP-1)/BMP-7, and Growth differentiation factor 5 (GDF-5)), transforming growth factor-β, insulin-like growth factor-1, and fibroblast growth factor have been investigated for their potential for biological repair and have been shown to elicit an anabolic effect on IVD cells [10-13]. A single injection of OP-1 [14-16] or GDF-5 [10] into the degenerative disc has been demonstrated to be reparative to aberrant discal matrices *in vivo*. However, concerns still remain about these recombinant human growth factors, including undesired blood vessel ingrowth in the IVD and the need to provide supraphysiologic doses to obtain effectiveness. Moreover, the high cost of these recombinant proteins can be prohibitive.

Intriguingly, Mundy and colleagues [17] conducted an examination of more than 30,000 compounds to determine their effect on expression of the BMP-2 gene. The 3-hydroxy-3-methylglutaryl coenzyme A reductase inhibitor statin, was the only compound that specifically increased BMP-2 mRNA in bone cells *in vitro *and subsequent bone formation *in vivo*. Our previous work initially illustrated that simvastatin stimulated endogenous BMP-2 expression in rat IVD cells cultured *in vitro*, which in turn promoted expression of a chondrogenic phenotype [18]. The present study serves to further test the impact of simvastatin on the degenerative disc in a defined animal model. It has been recognized that needle puncture can induce mild and progressive disc degeneration that is suitable for testing potential treatments for degenerative disc disease [19,20]. In this study, we used a disc degeneration model developed from our previous work, which can reproduce disc degeneration in rat tails consistently with less invasive perturbation [21]. The simvastatin-loaded compound was injected intradiscally and the effect of simvastatin on the degenerative disc was evaluated by changes in magnetic resonance imaging (MRI), gene expression, and biochemical and histological assays.

## Materials and methods

### Animals

Sprague-Dawley rats (three months old) were obtained from Harlan Laboratories (Indianapolis, IN, USA) and were housed in groups of three rats per cage. As rats reach their skeletal maturity before 3 months of age, the concern of IVD remodeling due to growth can be eliminated for the selected rats at the predetermined age used in this study [22,23]. Experiments were performed in accordance with the Guide for the Care and Use of Laboratory Animals, and the experimental protocols were approved by the University Committee on the Use and Care of Animals at the University of Michigan.

### Surgical technique

The surgical procedure described previously was followed in this study [21]. Briefly, anesthesia for all surgical procedures was achieved and maintained by inhalation of anesthetic isoflurane and the operative field was prepared in a sterile fashion. After palpation to determine the disc levels, a small skin cross-incision was made to help locate disc position for needle insertion. Co6/Co7 remained undisturbed as the control level. Fluoroscopy was used to visualize needle penetration and to ensure that the stab went into the center of the NP. A 21-gauge (G) needle was then inserted in the middle of the appropriate disc, controlled by a locking forceps clamped at 5 mm from the needle, through the AF into the NP of Co5/Co6 and Co7/Co8, rotated 180°, and held for five seconds.

### Simvastatin treatment

Four weeks after stab injury using the 21-G needle, either poly(ethylene glycol)-poly(lactic acid-co-glycolic acid)-poly(ethylene glycol) (PEG-PLGA-PEG) gel loaded with 2 μL of simvastatin (LKT Laboratories, St. Paul, MN, USA) or gel alone was slowly injected into the NP of Co5/Co6 and Co7/Co8 randomly using a microsyringe attached to a 31-G needle. The drug-loaded compound contained simvastatin at a concentration of 5 mg/mL. The 31-G needle was chosen for drug injection based on our trial results, because it does not cause injury leading to disc degeneration. The tri-block PEG-PLGA-PEG polymer was chosen as the drug vehicle, because it has the desired properties of being thermo-sensitive, biodegradable, biocompatible, and injectable, and it has been demonstrated to extend the duration of drug exposure [24,25]. For all subsequent experiments, four experiment groups were generated: the intact control group (without needle puncture, without gel injection); the stabbed group (with needle puncture, without gel injection); the gel-alone treatment group (with needle puncture, gel only injection); and the simvastatin treatment group (with needle puncture, gel loaded with simvastatin injection). At two weeks after the injection of gel loaded with or without simvastatin, the approached caudal spine levels of rat tails were assessed.

### Quantitative real-time PCR

Total RNA was extracted from the NP using the Trizol (Invitrogen, Carlsbad, CA, USA) reagent followed by RNeasy Mini Kit (Qiagen, Inc., Valencia, CA, USA). Reverse transcription was carried out at 42°C for 50 minutes using the SuperScript First-Strand Synthesis Kit (Invitrogen, Carlsbad, CA, USA). Type II collagen, type I collagen, aggrecan, BMP-2, and glyceraldehyde 3-phosphate dehydrogenase (GAPDH) gene expression were quantified by real-time PCR using Gene Amp 7700 Sequence Detection System (Applied Biosystems, Foster City, CA, USA). A positive standard curve for each primer was obtained by real-time PCR with serially-diluted cDNA sample mixture. Quantities of gene expression of BMP-2, aggrecan, and type I and type II collagens were calculated with standard samples and normalized with GAPDH. The amounts of mRNA expression were presented as a ratio to the intact control group.

### MRI procedures and data processing

At two weeks after injection of either gel loaded with simvastatin or gel alone, the approached caudal spine levels of rat tails were assessed by MRI. Briefly, rats were anesthetized with a 2% isoflurane/oxygen mixture throughout the entire MRI examination. The animal was laid prone and the tail was straightened in a 7.0 T Varian MR scanner (183 mm horizontal bore; Varian, Inc., Palo Alto, CA, USA), and body temperature was maintained at 37°C using circulating heated air. A double-tuned volume radiofrequency coil was used to scan the tail region of the rats. Eight serial T2-weighted sagittal images covering the entire disc area were acquired using a spin-echo sequence with the following parameters: fat saturation on; repetition time/effective echo time, 3000/30 ms; field of view, 30 × 60 mm; matrix, 128 × 128; slice thickness, 0.5 mm; slice spacing, 0 mm; number of slices, eight; number of scans, 1 (total scan time 6.24 minutes).

A procedure described previously was followed to quantitatively analyze the obtained image slices using Analyze 7.0 software (AnalyzeDirect, Overland Park, KS, USA) [21]. The nucleus region was segmented from the sliced images, followed by image reconstruction and volume rendering procedures to generate volumetric images and therefore calculate each nuclear volume. All image assessments described were conducted by three independent, blinded observers and the quantitative data were presented as the mean of the three evaluations. T2-weighted density as well as MRI index (the area of NP multiplied by average signal intensity) were quantified and calculated. For the MR index calculation, the NP area was defined by an image threshold automatically assigned by an image analysis software Analyze 7.0, which detects the default image densities over the acquired regions.

### Biochemical analyses

After discs were isolated from each level, a small incision was created in the AF by a sharp scissor and the entire NP (the gelatinous tissue) was taken out carefully using a micro scoop (Circon MicroSurgical, Santa Barbara, CA, USA). The dissected NP was weighed using an electronic scale, and digested with papain (125 μg/mL in sterile PBS with 5 mM cysteine. HCl and 5 mM Na_2_EDTA, pH 6.0) at 60°C for 24 hours. The proteoglycan, mainly sulfated glycosaminoglycan (sGAG), and the DNA contents in the digested solution were assayed using the dimethyl-methylene blue method [26] and Hoechst dye 33258 method [27], respectively.

### Histological analysis

After the animals were euthanized, three to four discs from each group were harvested for histological studies. Each disc, with 3.5 ± 0.5 mm of the adjacent vertebral bodies, was fixed in 10% neutral-buffered formalin for one week, decalcified in 22.5% formic acid and 10% sodium citrate for approximately two to three days. They were then processed for paraffin embedding and sectioning into sagittal sections (10 μm thick) using a microtome. Sections were stained with H&E and safranin O with fast-green counterstaining. Histological images were analyzed qualitatively under a light microscope (Olympus BX51; Olympus, Center Valley, PA, USA) at magnifications ranging from 4× to 100× to investigate changes in NP, AF, and endplates. The histological sections were also graded by blinded observers using the scoring standard established by Masuda and colleagues [19].

### Statistical analysis

All data were expressed as mean ± standard error. The significance of differences among the means of data for gene expression, MRI measurement, biochemical parameters, and histological scores were analyzed using one-way analysis of variance and Fisher's least significant difference as a *post hoc *test. A *P*-value of less than 0.05 was considered statistically significant.

## Results

### mRNA expression of BMP-2

**Figure 1:**
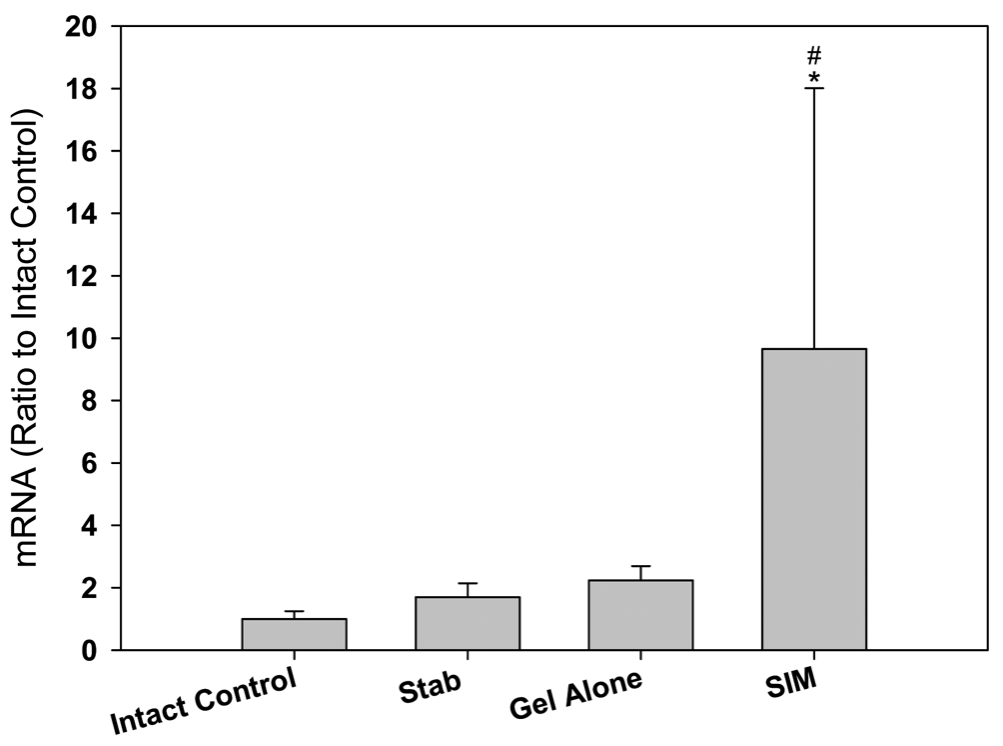
BMP-2 mRNA expression in the nucleus pulpous of different experimental groups at two weeks after drug injection. Discs in the treatment groups were injected with either 2 μL of gel alone or gel loaded with simvastatin (SIM; 5 mg/mL) at four weeks after disc stab with 21-G needle. * *P *< 0.05 when compared with intact control group,^# ^*P *< 0.05 when compared with stabbed group. BMP = Bone morphogenetic protein.

Figure 1 illustrates that BMP-2 mRNA expression in the NP among the intact control, stabbed, and gel-alone-treated groups was not significantly different. When rat discs were treated with simvastatin, BMP-2 mRNA expression was significantly increased compared with that of the intact control and stabbed groups (*P *< 0.05).

### mRNA expression of aggrecan, type I, and type II collagens

**Figure 2:**
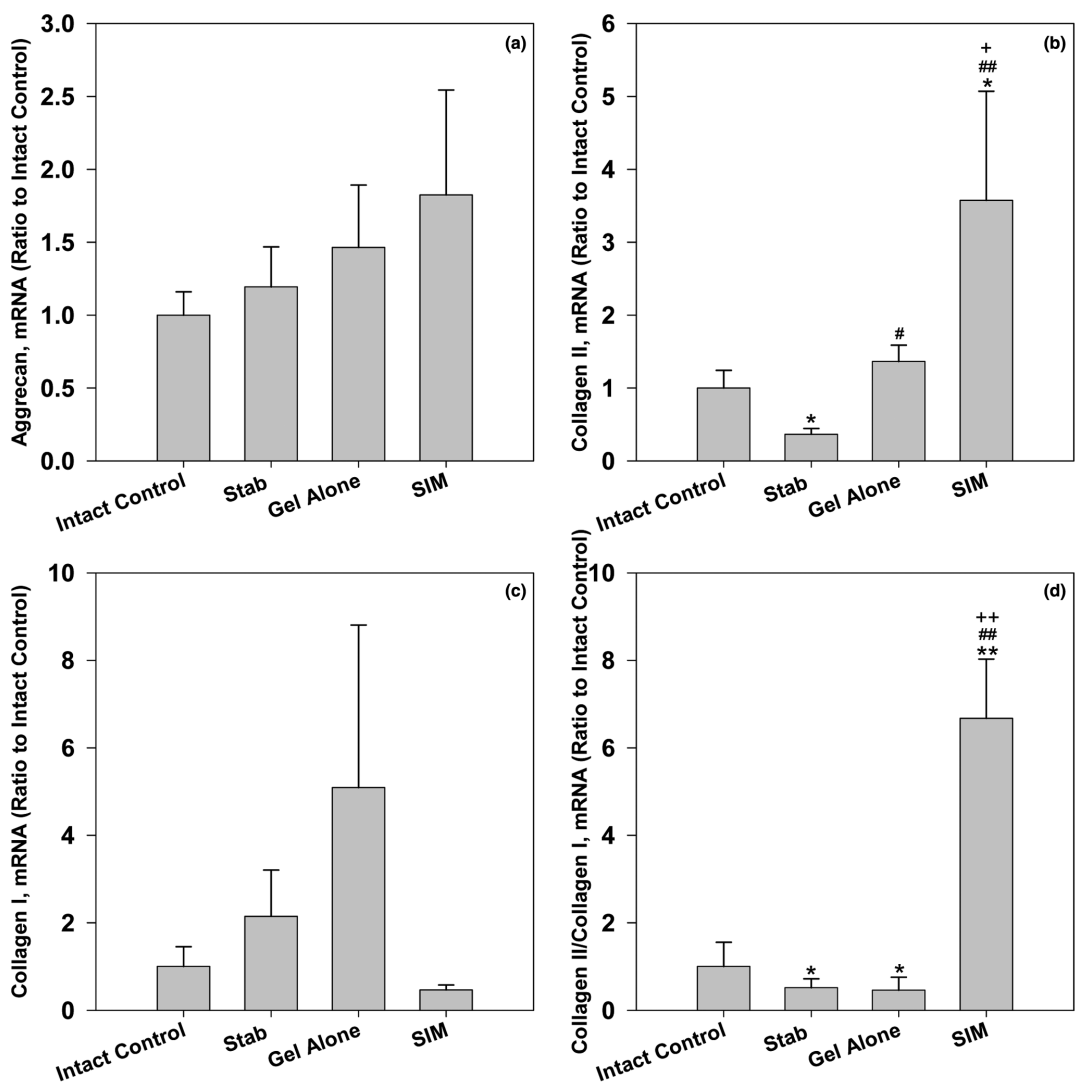
mRNA expression of **(a)** aggrecan, **(b)** type II collagen, **(c)** type I collagen, and **(d)** differentiation index in the nucleus pulpous of different experimental groups at two weeks after drug injection. The differentiation index is the ratio of type II to type I collagens. Discs in the treatment groups were injected with either 2 μL of gel alone or gel loaded with simvastatin (SIM; 5 mg/mL) at four weeks after the disc stab with 21-G needle. * *P *< 0.05 and ** *P *< 0.01 when compared with intact control group;^# ^*P *< 0.05 and ^## ^*P *< 0.01 when compared with stabbed group; ^+ ^*P *< 0.05 and ^++ ^*P *< 0.01 when compared with gel-alone-treated group.

Groups treated with simvastatin showed an increase in mRNA expression of aggrecan although the increase did not reach significance (Figure 2a). However, the mRNA level of type II collagen, another key phenotypical molecule of chondrogenesis, was significantly lower in the stabbed discs when compared with the intact ones (*P *< 0.05). When discs were treated with gel alone, the mRNA level of type II collagen changed significantly compared with that of the stabbed discs (*P *< 0.05). The mRNA level of type II collagen was most elevated in discs treated with the compound loaded with simvastatin. Statistical analysis showed that collagen type II mRNA expression was significantly increased in the simvastatin-treated groups when compared with the other three groups (Figure 2b; *P *< 0.05 when compared with intact control and gel-alone-treated group, and *P *< 0.01 when compared with stabbed group).

The mRNA expression of type I collagen that typically reflects dedifferentiation of chondrocytes was also measured in our study. The mRNA levels of type I collagen in the stabbed disc and those treated with gel alone presented an increase at two weeks after injection. When the stabbed disc was treated with simvastatin, the mRNA level of type I collagen in this group, on the other hand, was decreased. However, *post hoc *comparisons indicated there was no significant difference among all experimental groups (Figure 2c).

The ratio of type II to type I collagens is typically referred to as a 'differentiation index' of chondrocytes to demonstrate the propensity for chondrogenesis. As shown in Figure 2d, the differentiation index in the stabbed control group was significantly decreased when compared with that of the intact control group (*P *< 0.05). There was no significant difference between the differentiation indices of the stabbed control and gel-treated groups. However, the index of the simvastatin-treated group was significantly higher than all other groups at time of the investigation (*P *< 0.01).

### MRI assessment

**Figure 3:**
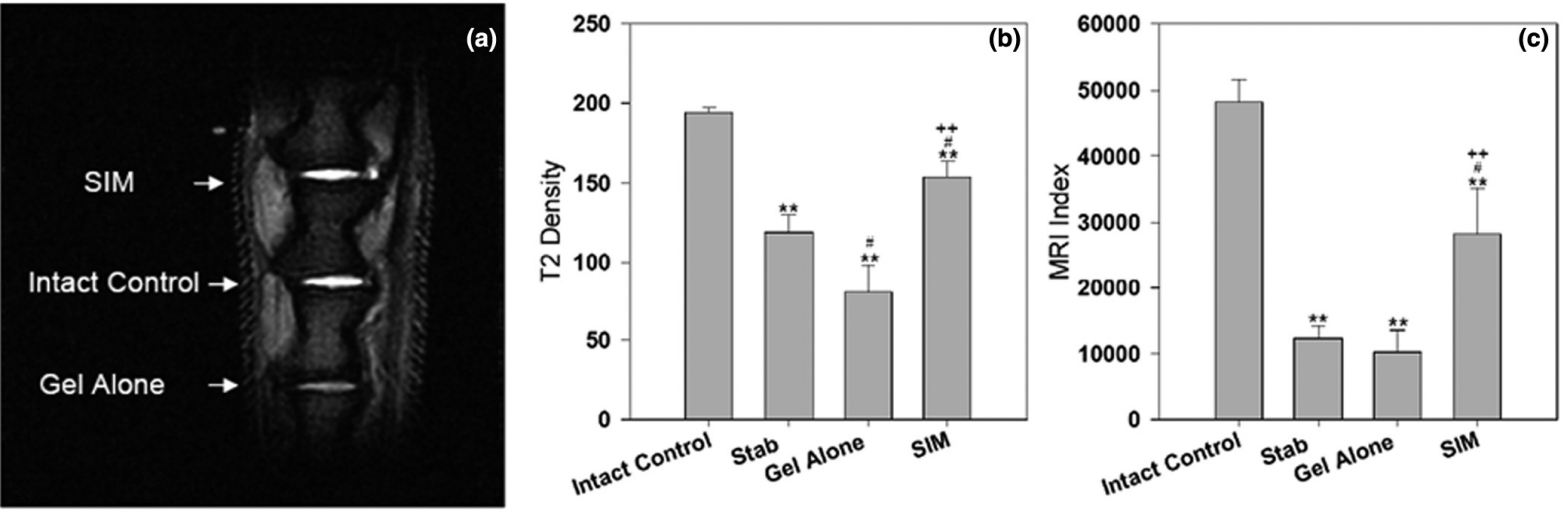
Representative MRI scans and quantitative analysis of T2 density and MRI index of different experimental groups at two weeks after drug injection. Discs in treatment groups were injected with either 2 μL of gel alone or gel loaded with simvastatin (SIM; 5 mg/mL) at four weeks after the disc stab with 21-G needle. ** *P *< 0.01 when compared with intact control group;^# ^*P *< 0.05 when compared with stabbed group; ^++ ^*P *< 0.01 when compared with gel-alone-treated group. MRI = magnetic resonance imaging.

Representative T2-weighted, midsagittal images of the approached rat caudal disc are shown in Figure 3. At two weeks after injection, MRIs of the NP in the simvastatin-treated group showed stronger signal intensities than those in the stabbed group and the group treated with hydrogel alone (Figure 3a). When compared with the intact control group, the T2 density and MRI index of the stabbed group were significantly decreased (*P *< 0.01). T2 density in the gel-alone-treated group showed a significant decrease when compared with the stabbed group (*P *< 0.05). However, no significant change in MRI index was noticed between the stabbed and the gel-treated groups. In the simvastatin-treated group, the T2 signals and MRI indices in the perturbed discs were all significantly increased at two weeks after injection compared with those of the stabbed group (*P *< 0.05) and the gel-alone-treated group (*P *< 0.01; Figures 3b and 3c).

### Biochemical analyses

**Figure 4:**
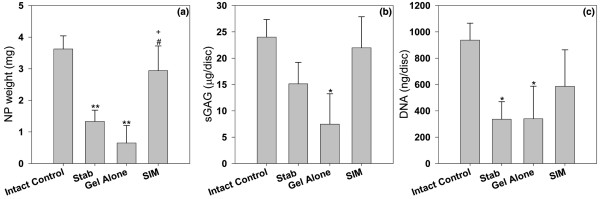
Weight, sGAG, and DNA content in the NP of different experimental groups at two weeks after drug injection. Discs in the treatment groups were injected with either 2 μL of gel alone or gel loaded with simvastatin (SIM; 5 mg/mL) at four weeks after disc stab with 21-G needle. * *P *< 0.05 and ** *P *< 0.01 when compared with intact control group;^# ^*P *< 0.05 when compared with stabbed group; ^+ ^*P *< 0.05 when compared with gel-alone-treated group. NP = nucleus pulpous; sGAG = glycosaminoglycan.

The weight of a NP normally decreases with progression of disc degeneration. The weight of the NP in the intact control group was 3.8 ± 0.33 mg/disc. It dropped to 1.72 ± 0.24 mg/disc in the stabbed control group, which showed a significant difference compared with intact controls (*P *< 0.01). No significant difference was noticed between the stabbed and the gel-alone-treated groups. After stabbed discs were treated with simvastatin, the weight of the NP significantly increased compared with the stabbed control group and gel-alone-treated group (*P *< 0.05; Figure 4a).

The sGAG content in the stabbed groups was lower than that in the intact control group. The difference, however, was not statistically significant between the two groups. The sGAG content in the gel-alone-treated discs decreased significantly as compared with the intact control group (*P *< 0.05). Treatment with simvastatin increased sGAG content in the stabbed discs; however, the increase did not reach statistical significance (Figure 4b).

To determine whether simvastatin cell viability, the DNA content in the affected NP was measured. As shown in Figure 4c, DNA content in the stabbed and gel-alone-treated groups was significantly decreased compared with that of the intact control (*P *< 0.05). Two weeks of simvastatin treatment resulted in an increase in the DNA content that had decreased due to needle puncture. *Post hoc *analysis did not show a significant difference between the simvastatin-treated group and gel-alone-treated group.

### Histological changes

**Figure 5:**
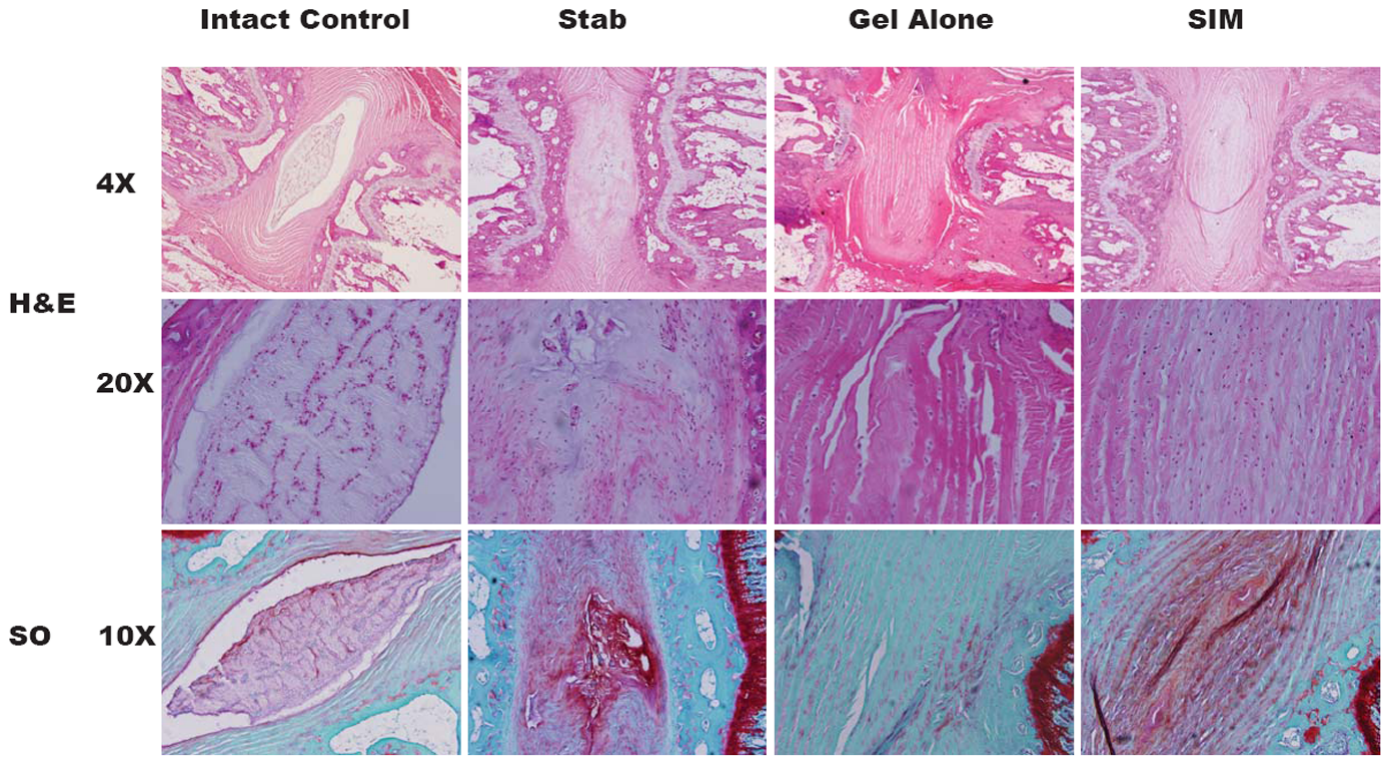
Representative H&E (4× and 20×) and safranin O stainings (10×) of disc samples from different experimental groups at two weeks after drug injection. Discs in the treatment groups were treated with either 2 μL of gel alone or gel loaded with simvastatin (SIM; 5 mg/mL) at four weeks after disc stab with 21-G needle. SO = safranin O.

In the intact disc stained by H&E, the boat-shaped NP comprised at least one-half of the disc area at the midsagittal cross-section. The AF was intact and the border between the AF and the NP was clearly defined. In the stabbed group, NP area in the 21-G needle-stabbed disc decreased and became irregular, with some areas densely staining. Discs in the gel-alone-treated group still displayed features of disc degeneration to a certain extent. Conversely, the cell distribution and the extracellular matrix alignment in the 21 G-needle stabbed animals treated with simvastatin were more even and regular than those in the stab and gel-alone groups. The NP area in the intact disc stained strongly with safranin O. In the stabbed discs, the positively stained area decreased. However, the stained levels were not homogenous, as some regions stained very densely compared with surrounding vicinities. The positively stained area in the gel-alone-treated group was much smaller and weaker. When the disc was treated with simvastatin, the NP area was stained more positively than those in the gel-alone-treated group (Figure 5).

**Figure 6:**
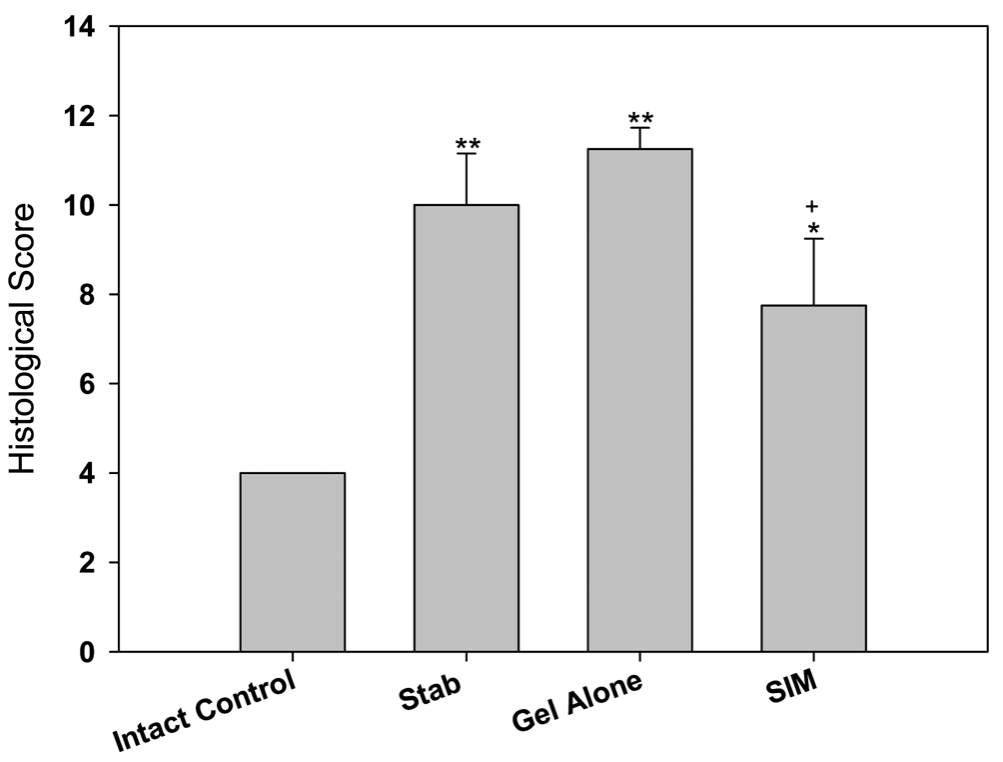
Histological scores of different experimental groups at two weeks after drug injection. Discs in the treatment groups were injected with either 2 μL of gel alone or gel loaded with simvastatin (SIM; 5 mg/mL) at four weeks after disc stab with 21-G needle. Changes in histological appearance were assessed semi-quantitatively. * *P *< 0.05 and ** *P *< 0.01 when compared with intact control group;^+ ^*P *< 0.05 when compared with gel-alone-treated group.

Semi-quantitative histological scores were 4 ± 0 for intact control, 10 ± 1.15 for stabbed control, 11.25 ± 0.48 for gel-alone-treated group, and 7.75 ± 1.49 for simvastatin-treated group. Statistical analysis showed the histological scores of the stabbed discs in the stabbed and gel-alone-treated groups significantly increased when compared with intact control discs (*P *< 0.01). There was no significant difference in histological scores between the stabbed and gel-alone-treated groups. The histological score of the simvastatin-treated group, however, was significantly lower than the gel-alone-treated group (*P *< 0.05, Figure 6).

## Discussion

IVD degeneration is mostly characterized by changes in disc morphology and composition of the extracellular matrix, as well as loss of disc cells and water content. To achieve optimal disc repair, the ideal therapy should preserve the intact architecture of the disc tissue as much as possible, while increasing the synthesis of water-absorbing molecules, such as type II collagen and proteoglycan, to restore its 'shock-cushion' function. Most of the new biology-based strategies for disc repair are based upon these principles, including autologous cell transplantation, growth factor injection, or gene delivery. The present study provides a promising alternative that is potentially more easily translated to the clinical setting. Our results show that a single dose of simvastatin loaded in PEG-PLGA-PEG gel injected into the NP of rat discs up-regulates mRNA levels of collagen type II and the differentiation index (the ratio of type II to type I collagen). The improvements were also evident in the changes in NP weight, histological morphology, and MRI at certain significant levels.

Our previous study demonstrated that simvastatin promoted expression of a chondrogenic phenotype of rat IVD cells, mainly through the up-regulated BMP-2-mediated pathway [18]. To determine if the stimulation of BMP-2 mRNA expression can be replicated *in vivo *by simvastatin, gene expression of BMP-2 in NP tissue was initially measured. The results indicated that injection of simvastatin up-regulated BMP-2 mRNA levels, thus promoting an anabolic mechanism for IVD repair. Earlier studies have also proposed a similar concept to facilitate IVD repair, although the methods involved either direct exogenous supplementation of BMP-2 [12,28,29] or endogenous up-regulation of BMP-2 with the over-expression of LIM mineralization protein-1 [30].

The anabolic stimulus primarily included significant increases in mRNA expression of type II collagen, and the ratio of type II to type I collagen. Previous studies indicated an increase in type I collagen and a reduction in type II collagen were present in the degenerating NP [31]. The 'differentiation index', which is defined by the ratio of gene expression of type II to type I collagen, has become a method to track the functionality of chondrocytes (or chondrocyte-like cells in the case of NP) and can be used as a reference to measure degeneration of cartilaginous tissues [32].

One interesting observation from our results involved chondrogenic expression stimulated by the delivered simvastatin. When normal IVD cells were treated with simvastatin, the mRNA expression of both aggrecan and type II collagen were significantly up-regulated [18], whereas only the mRNA level of type II collagen in the degenerative NP increased after simvastatin treatment in the present *in vivo *study. Due to the changes in the notochord cells during the process of disc degeneration [33] and the different susceptibility to the stimuli between notochord cells and chondrocyte-like cells [34], the discrepancy in the responsiveness of normal and degenerative rat IVD cells to simvastatin may imply that the degree of disc degeneration and/or the cell components may affect the response of treated cells. Further studies are required to explore more details before any conclusions can be drawn.

The efficacy of simvastatin was also demonstrated by changes in sGAG content, MRI analysis, and NP weight. It should be highlighted that although the MRI and histological assays showed evidence of disc degeneration in the stabbed group, the sGAG content of this group was not significantly reduced compared with the intact control. This phenomenon has been observed in previous studies, and has been attributed to condensation of the extracellular matrix as an early response to the onset of disc degeneration [21,23,35]. In the group treated with gel alone, none of the measurements were improved. Conversely, the MRI, histological assessment, and NP weight were significantly improved in the simvastatin-treated group.

Our previous study raised the concern of decreased cell viability, despite the significant augmentation of chondrogenesis in the IVD cells by simvastatin *in vitro *[18]. In the present study, a similar finding was not observed according to DNA analysis. It is likely that the use of PEG-PLGA-PEG hydrogel to deliver simvastatin minimized the likelihood of exposing cells to a transiently high concentration of the drug at the time of injection. This tri-block polymer has been used extensively as a carrier for controlled drug or gene delivery [24,36,37]. Our pilot data in a separate study also demonstrated that the release profile of simvastatin from the PEG-PLGA-PEG gel *in vitro *was steady and sustainable (data not shown). In addition, the polymer was chosen for its property of sol-gel phase transition, and slow degradation [38]. After simvastatin is incorporated into the liquid gel and injected into the NP, the compound rapidly converts to the gel phase as soon as it reaches body temperature. Within the gel format, it would be expected that the exposure of simvastatin to the IVD cells can be confined to the discal core and the delivery can be sustained [39]. The safety concern of the gel is likely to be minimal, because PEG-PLGA-PEG hydrogel has been well characterized and is currently undergoing phase I clinical trials for the treatment of breast cancer [40].

It should be noted that a recent study showed different results by introducing BMP-2 intradiscally for 12 weeks in a rabbit degenerative disc model. In that study, the discs treated with BMP-2 developed degenerative changes [41]. The differing results may reflect the fact that the delivered dose and the induced concentration of BMP-2 were quite different. The direct injection of 0.1 mg of BMP-2 was supraphysiological compared with the endogenous level of BMP-2 induced by simvastatin in our study. It has been reported that a dose of 0.1 mg of BMP-2 is in the optimal range for successful posterolateral fusion in rabbits [42]. However, for achieving cartilage repair, BMP-2 was used at lower doses (1 to 15 μg) [43-45] or by over-expression of BMP-2 using a virus vector [46]. In our previous study, the amount of induced BMP-2 (60 to 140 pg/mL) was sufficient to promote chondrogenic expression in the IVD cells (unpublished publication). Similarly, the results of this study suggest that the concentration of induced BMP-2 by simvastatin treatment is in the therapeutic range to achieve disc repair. The degree of disc degeneration may have also resulted in the conflicting observed responses to BMP-2 stimuli. In the rabbit study by Huang and colleagues [41], the model used to simulate disc degeneration involved a full-thickness lesion created by blade penetration, whereas a mild and slower progression of disc degeneration occurred in the stabbed disc method used in our study. It has been shown that the BMP-2 receptor BMPRII was expressed predominantly in the AF associated with severe disc degeneration in our previous study [21], and the BMP-2 uptake at this site (where fibroblast-like cells reside) typically leads to osteophyte formation, because it has been shown in several studies that exogenous BMP-2 induced the BMPRII expression of ligamentous fibroblasts and, in turn, initiated endochondral ossification [47,48].

In addition to anabolism of chondrogenesis by simvastatin, there is evidence that statins can elicit anti-inflammatory actions in activated human chondrocytes [49], IL-1β-stimulated human chondrocytes [50], and experimental osteoarthritis in rabbits [51] by inhibiting the level of matrix metalloproteinase (MMP). For disc cells, increased MMPs have been found to promote disc degeneration [52-54]. A recent study even specified that the biochemical mediators of inflammation and tissue degradation including MMP-3, IL-1β and inducible nitric oxide synthase were significantly increased along with the disc degeneration that was induced by needle puncture [55]. Of interest is whether simvastatin can also help suppress catabolism of chondrogenesis in degenerative disc, and thus, is the focus of our next study.

## Conclusions

Local injection of a simvastatin-loaded PEG-PLGA-PEG compound was preliminarily found to promote autogenous chondrogenic disc repair and retard disc degeneration, which provides an alternative strategy for biological disc repair in a less expensive and easily applied method. Due to the limitation that the rat model may differ from the scheme of human disc degeneration, particularly in biomechanical functions and cellular components, future research including a long-term treatment study in this animal model as well as in a large animal model will be required to demonstrate more definitively that intradiscal injection of simvastatin would be useful for the retardation of disc degeneration.

## Abbreviations

AF: annulus fibrosus; BMP: bone morphogenetic protein; G: gauge; GAPDH: glyceraldehyde 3-phosphate dehydrogenase; H&E: hematoxylin and eosin; IL-1β: interleukin-1β; IVD: intervertebral disc; MMP: matrix metalloproteinase; MRI: magnetic resonance imaging; NP: nucleus pulpous; PBS: phosphate-buffered saline; PCR: polymerase chain reaction; PEG-PLGA-PEG: poly(ethylene glycol)-poly(lactic acid-co-glycolic acid)-poly(ethylene glycol); sGAG: glycosaminoglycan.

## Competing interests

A provisional patent related to the content of the manuscript was disclosed in January 2008 and currently the utility patent following this disclosure has been prepared and filed. The ownership of the stated patent belongs to the University of Michigan and no kind of financial aids have been received to support the related research efforts.

## Authors' contributions

HZ designed, carried out the entire study, participated in the acquisition of data, analyzed and interpreted data, and drafted the manuscript. LW participated in the acquisition and interpretation of the histological data. PJB performed the gel processing and drug preparation for injection. PP, VCY, SJH, FLM conceived the study and performed critical review and revision of the manuscript. CYL conceived the study, helped secure funding, revised the manuscript, and gave final approval of the version to be submitted. All authors read and approved the final manuscript.

## References

[B1] Rasmussen S Krum-Moller DS Lauridsen LR Jensen SE Mandoe H Gerlif C Kehlet H Epidural steroid following discectomy for herniated lumbar disc reduces neurological impairment and enhances recovery: a randomized study with two-year follow-up Spine 2008 33 2028 2033 10.1097/BRS.0b013e3181833903 18758356

[B2] Buttermann GR The effect of spinal steroid injections for degenerative disc disease Spine J 2004 4 495 505 10.1016/j.spinee.2004.03.024 15363419

[B3] Olah M Molnar L Dobai J Olah C Feher J Bender T The effects of weightbath traction hydrotherapy as a component of complex physical therapy in disorders of the cervical and lumbar spine: a controlled pilot study with follow-up Rheumatol Int 2008 28 749 756 10.1007/s00296-008-0522-y 18193231

[B4] Saunders HD Use of spinal traction in the treatment of neck and back conditions Clin Orthop Relat Res 1983 179 31 38 10.1097/00003086-198310000-00006 6617030

[B5] Wetzel FT McNally TA Phillips FM Intradiscal electrothermal therapy used to manage chronic discogenic low back pain: new directions and interventions Spine 2002 27 2621 2626 10.1097/00007632-200211150-00043 12436005

[B6] Pomerantz SR Hirsch JA Intradiscal therapies for discogenic pain Semin Musculoskelet Radiol 2006 10 125 135 10.1055/s-2006-939030 16586321

[B7] Zhang Y An HS Tannoury C Thonar EJ Freedman MK Anderson DG Biological treatment for degenerative disc disease: implications for the field of physical medicine and rehabilitation Am J Phys Med Rehabil 2008 87 694 702 10.1097/PHM.0b013e31817c1945 18716481

[B8] Masuda K An HS Prevention of disc degeneration with growth factors Eur Spine J 2006 15 Suppl 3 S422 432 10.1007/s00586-006-0149-1 16865380 PMC2335371

[B9] Masuda K Biological repair of the degenerated intervertebral disc by the injection of growth factors Eur Spine J 2008 17 Suppl 4 441 451 10.1007/s00586-008-0749-z 19005698 PMC2587664

[B10] Chujo T An HS Akeda K Miyamoto K Muehleman C Attawia M Andersson G Masuda K Effects of growth differentiation factor-5 on the intervertebral disc--in vitro bovine study and in vivo rabbit disc degeneration model study Spine 2006 31 2909 2917 10.1097/01.brs.0000248428.22823.86 17139221

[B11] Gruber HE Fisher EC Jr Desai B Stasky AA Hoelscher G Hanley EN Jr Human intervertebral disc cells from the annulus: three-dimensional culture in agarose or alginate and responsiveness to TGF-beta1 Exp Cell Res 1997 235 13 21 10.1006/excr.1997.3647 9281347

[B12] Li J Yoon ST Hutton WC Effect of bone morphogenetic protein-2 (BMP-2) on matrix production, other BMPs, and BMP receptors in rat intervertebral disc cells J Spinal Disord Tech 2004 17 423 428 10.1097/01.bsd.0000112084.85112.5d 15385883

[B13] Osada R Ohshima H Ishihara H Yudoh K Sakai K Matsui H Tsuji H Autocrine/paracrine mechanism of insulin-like growth factor-1 secretion, and the effect of insulin-like growth factor-1 on proteoglycan synthesis in bovine intervertebral discs J Orthop Res 1996 14 690 699 10.1002/jor.1100140503 8893760

[B14] Masuda K Imai Y Okuma M Muehleman C Nakagawa K Akeda K Thonar E Andersson G An HS Osteogenic protein-1 injection into a degenerated disc induces the restoration of disc height and structural changes in the rabbit anular puncture model Spine 2006 31 742 754 10.1097/01.brs.0000206358.66412.7b 16582847

[B15] Imai Y Okuma M An HS Nakagawa K Yamada M Muehleman C Thonar E Masuda K Restoration of disc height loss by recombinant human osteogenic protein-1 injection into intervertebral discs undergoing degeneration induced by an intradiscal injection of chondroitinase ABC Spine 2007 32 1197 1205 10.1097/BRS.0b013e3180574d26 17495776

[B16] Chubinskaya S Kawakami M Rappoport L Matsumoto T Migita N Rueger DC Anti-catabolic effect of OP-1 in chronically compressed intervertebral discs J Orthop Res 2007 25 517 530 10.1002/jor.20339 17205567

[B17] Mundy G Garrett R Harris S Chan J Chen D Rossini G Boyce B Zhao M Gutierrez G Stimulation of bone formation in vitro and in rodents by statins Science 1999 286 1946 1949 10.1126/science.286.5446.1946 10583956

[B18] Zhang H Lin CY Simvastatin stimulates chondrogenic phenotype of intervertebral disc cells partially through BMP-2 pathway Spine 2008 33 E525 531 10.1097/BRS.0b013e31817c561b 18628692

[B19] Masuda K Aota Y Muehleman C Imai Y Okuma M Thonar EJ Andersson GB An HS A novel rabbit model of mild, reproducible disc degeneration by an anulus needle puncture: correlation between the degree of disc injury and radiological and histological appearances of disc degeneration Spine 2005 30 5 14 15626974 10.1097/01.brs.0000148152.04401.20

[B20] Sobajima S Kompel JF Kim JS Wallach CJ Robertson DD Vogt MT Kang JD Gilbertson LG A slowly progressive and reproducible animal model of intervertebral disc degeneration characterized by MRI, X-ray, and histology Spine 2005 30 15 24 15626975 10.1097/01.brs.0000148048.15348.9b

[B21] Zhang H La Marca F Hollister SJ Goldstein SA Lin C-Y Developing consistently reproducible intervertebral disc degeneration at rat caudal spine by using needle puncture J Neurosurg: Spine 2009 10 522 530 10.3171/2009.2.SPINE08925 19558284

[B22] Hughes PC Tanner JM The assessment of skeletal maturity in the growing rat J Anat 1970 106 371 402 4315144 PMC1233709

[B23] Rousseau MA Ulrich JA Bass EC Rodriguez AG Liu JJ Lotz JC Stab incision for inducing intervertebral disc degeneration in the rat Spine 2007 32 17 24 10.1097/01.brs.0000251013.07656.45 17202887

[B24] Tyagi P Li Z Chancellor M De Groat WC Yoshimura N Huang L Sustained intravesical drug delivery using thermosensitive hydrogel Pharm Res 2004 21 832 837 10.1023/B:PHAM.0000026436.62869.9c 15180342

[B25] Chang CW Choi D Kim WJ Yockman JW Christensen LV Kim YH Kim SW Non-ionic amphiphilic biodegradable PEG-PLGA-PEG copolymer enhances gene delivery efficiency in rat skeletal muscle J Control Release 2007 118 245 253 10.1016/j.jconrel.2006.11.025 17270304

[B26] Chandrasekhar S Esterman MA Hoffman HA Microdetermination of proteoglycans and glycosaminoglycans in the presence of guanidine hydrochloride Anal Biochem 1987 161 103 108 10.1016/0003-2697(87)90658-0 3578776

[B27] Kim YJ Sah RL Doong JY Grodzinsky AJ Fluorometric assay of DNA in cartilage explants using Hoechst 33258 Anal Biochem 1988 174 168 176 10.1016/0003-2697(88)90532-5 2464289

[B28] Tim Yoon S Su Kim K Li J Soo Park J Akamaru T Elmer WA Hutton WC The effect of bone morphogenetic protein-2 on rat intervertebral disc cells in vitro Spine 2003 28 1773 1780 10.1097/01.BRS.0000083204.44190.34 12923462

[B29] Kim DJ Moon SH Kim H Kwon UH Park MS Han KJ Hahn SB Lee HM Bone morphogenetic protein-2 facilitates expression of chondrogenic, not osteogenic, phenotype of human intervertebral disc cells Spine 2003 28 2679 2684 10.1097/01.BRS.0000101445.46487.16 14673369

[B30] Yoon ST Park JS Kim KS Li J Attallah-Wasif ES Hutton WC Boden SD ISSLS prize winner: LMP-1 upregulates intervertebral disc cell production of proteoglycans and BMPs in vitro and in vivo Spine 2004 29 2603 2611 10.1097/01.brs.0000146103.94600.85 15564908

[B31] Nerlich AG Schleicher ED Boos N 1997 Volvo Award winner in basic science studies. Immunohistologic markers for age-related changes of human lumbar intervertebral discs Spine 1997 22 2781 2795 10.1097/00007632-199712150-00001 9431614

[B32] Yang KG Saris DB Verbout AJ Creemers LB Dhert WJ The effect of synovial fluid from injured knee joints on in vitro chondrogenesis Tissue Eng 2006 12 2957 2964 10.1089/ten.2006.12.2957 17518663

[B33] Yang F Leung VY Luk KD Chan D Cheung KM Injury-induced sequential transformation of notochordal nucleus pulposus to chondrogenic and fibrocartilaginous phenotype in the mouse J Pathol 2009 218 113 121 10.1002/path.2519 19288580

[B34] Guehring T Wilde G Sumner M Grunhagen T Karney GB Tirlapur UK Urban JP Notochordal intervertebral disc cells: sensitivity to nutrient deprivation Arthritis Rheum 2009 60 1026 1034 10.1002/art.24407 19333932

[B35] Lipson SJ Muir H Experimental intervertebral disc degeneration: morphologic and proteoglycan changes over time Arthritis Rheum 1981 24 12 21 10.1002/art.1780240103 7470167

[B36] Jeong B Bae YH Kim SW Drug release from biodegradable injectable thermosensitive hydrogel of PEG-PLGA-PEG triblock copolymers J Control Release 2000 63 155 163 10.1016/S0168-3659(99)00194-7 10640589

[B37] Li Z Ning W Wang J Choi A Lee PY Tyagi P Huang L Controlled gene delivery system based on thermosensitive biodegradable hydrogel Pharm Res 2003 20 884 888 10.1023/A:1023887203111 12817892

[B38] Jeong B Bae YH Kim SW In situ gelation of PEG-PLGA-PEG triblock copolymer aqueous solutions and degradation thereof J Biomed Mater Res 2000 50 171 177 10.1002/(SICI)1097-4636(200005)50:2<171::AID-JBM11>3.0.CO;2-F 10679681

[B39] Jeong B Bae YH Lee DS Kim SW Biodegradable block copolymers as injectable drug-delivery systems Nature 1997 388 860 862 10.1038/42218 9278046

[B40] Vukelja SJ Anthony SP Arseneau JC Berman BS Cunningham CC Nemunaitis JJ Samlowski WE Fowers KD Phase 1 study of escalating-dose OncoGel (ReGel/paclitaxel) depot injection, a controlled-release formulation of paclitaxel, for local management of superficial solid tumor lesions Anticancer Drugs 2007 18 283 289 10.1097/CAD.0b013e328011a51d 17264760

[B41] Huang KY Yan JJ Hsieh CC Chang MS Lin RM The in vivo biological effects of intradiscal recombinant human bone morphogenetic protein-2 on the injured intervertebral disc: an animal experiment Spine 2007 32 1174 1180 10.1097/01.brs.0000263369.95182.19 17495773

[B42] Itoh H Ebara S Kamimura M Tateiwa Y Kinoshita T Yuzawa Y Takaoka K Experimental spinal fusion with use of recombinant human bone morphogenetic protein 2 Spine 1999 24 1402 1405 10.1097/00007632-199907150-00003 10423783

[B43] Sellers RS Zhang R Glasson SS Kim HD Peluso D D'Augusta DA Beckwith K Morris EA Repair of articular cartilage defects one year after treatment with recombinant human bone morphogenetic protein-2 (rhBMP-2) J Bone Joint Surg Am 2000 82 151 160 10.1302/0301-620X.82B1.10733 10682724

[B44] Suzuki T Bessho K Fujimura K Okubo Y Segami N Iizuka T Regeneration of defects in the articular cartilage in rabbit temporomandibular joints by bone morphogenetic protein-2 Br J Oral Maxillofac Surg 2002 40 201 206 10.1054/bjom.2001.0720 12054709

[B45] Nawata M Wakitani S Nakaya H Tanigami A Seki T Nakamura Y Saito N Sano K Hidaka E Takaoka K Use of bone morphogenetic protein 2 and diffusion chambers to engineer cartilage tissue for the repair of defects in articular cartilage Arthritis Rheum 2005 52 155 163 10.1002/art.20713 15641068

[B46] Blaney Davidson EN Vitters EL van Lent PL Loo FA van de Berg WB van den Kraan PM van der Elevated extracellular matrix production and degradation upon bone morphogenetic protein-2 (BMP-2) stimulation point toward a role for BMP-2 in cartilage repair and remodeling Arthritis Res Ther 2007 9 R102 10.1186/ar2305 17922907 PMC2212581

[B47] Hoshi K Amizuka N Sakou T Kurokawa T Ozawa H Fibroblasts of spinal ligaments pathologically differentiate into chondrocytes induced by recombinant human bone morphogenetic protein-2: morphological examinations for ossification of spinal ligaments Bone 1997 21 155 162 10.1016/S8756-3282(97)00106-3 9267691

[B48] Onishi T Ishidou Y Nagamine T Yone K Imamura T Kato M Sampath TK ten Dijke P Sakou T Distinct and overlapping patterns of localization of bone morphogenetic protein (BMP) family members and a BMP type II receptor during fracture healing in rats Bone 1998 22 605 612 10.1016/S8756-3282(98)00056-8 9626398

[B49] Dombrecht EJ Van Offel JF Bridts CH Ebo DG Seynhaeve V Schuerwegh AJ Stevens WJ De Clerck LS Influence of simvastatin on the production of pro-inflammatory cytokines and nitric oxide by activated human chondrocytes Clin Exp Rheumatol 2007 25 534 539 17888208

[B50] Lazzerini PE Capecchi PL Nerucci F Fioravanti A Chellini F Piccini M Bisogno S Marcolongo R Laghi Pasini F Simvastatin reduces MMP-3 level in interleukin 1beta stimulated human chondrocyte culture Ann Rheum Dis 2004 63 867 869 10.1136/ard.2003.009746 15194586 PMC1755052

[B51] Akasaki Y Matsuda S Nakayama K Fukagawa S Miura H Iwamoto Y Mevastatin reduces cartilage degradation in rabbit experimental osteoarthritis through inhibition of synovial inflammation Osteoarthritis Cartilage 2009 17 235 243 10.1016/j.joca.2008.06.012 18672387

[B52] Goupille P Jayson MI Valat JP Freemont AJ Matrix metalloproteinases: the clue to intervertebral disc degeneration? Spine 1998 23 1612 1626 10.1097/00007632-199807150-00021 9682320

[B53] Weiler C Nerlich AG Zipperer J Bachmeier BE Boos N 2002 SSE Award Competition in Basic Science: expression of major matrix metalloproteinases is associated with intervertebral disc degradation and resorption Eur Spine J 2002 11 308 320 10.1007/s00586-002-0472-0 12193991 PMC3610483

[B54] Shen B Melrose J Ghosh P Taylor F Induction of matrix metalloproteinase-2 and -3 activity in ovine nucleus pulposus cells grown in three-dimensional agarose gel culture by interleukin-1beta: a potential pathway of disc degeneration Eur Spine J 2003 12 66 75 12592549 10.1007/s00586-002-0454-2

[B55] Sobajima S Shimer AL Chadderdon RC Kompel JF Kim JS Gilbertson LG Kang JD Quantitative analysis of gene expression in a rabbit model of intervertebral disc degeneration by real-time polymerase chain reaction Spine J 2005 5 14 23 10.1016/j.spinee.2004.05.251 15653081

